# The human papillomavirus (HPV) E7 protein antagonises an Imiquimod-induced inflammatory pathway in primary human keratinocytes

**DOI:** 10.1038/srep12922

**Published:** 2015-08-13

**Authors:** Kathryn H. Richards, Christopher W. Wasson, Oliver Watherston, Rosella Doble, G. Eric Blair, Miriam Wittmann, Andrew Macdonald

**Affiliations:** 1 School of Molecular and Cellular Biology, Astbury Centre for Structural Molecular Biology, Faculty of Biological Sciences, University of Leeds, Leeds, LS2 9JT, United Kingdom; 2 Leeds Institute of Rheumatic and Musculoskeletal Medicine, University of Leeds, Leeds, United Kingdom; 3 NIHR Leeds Musculoskeletal Biomedical Research Unit, Chapel Allerton Hospital Leeds, United Kingdom; 4 Centre for Skin Sciences, School of Life Sciences, University of Bradford, Bradford, United Kingdom

## Abstract

High-risk human papillomaviruses (HPV) are the etiological pathogen of cervical and a number of ano-genital cancers. How HPVs overcome the significant barriers of the skin immune system has been the topic of intensive research. The E6 and E7 oncoproteins have emerged as key players in the deregulation of host innate immune pathways that are required for the recruitment of effector cells of the immune response. Here we demonstrate that E7, and to a lesser extend E6, strongly reduce NFκB activation in response to the inflammatory mediator imiquimod. Moreover, we establish that undifferentiated keratinocytes do not express the putative receptor for imiquimod, TLR7, and as such are stimulated by imiquimod through a novel pathway. Inhibition of imiquimod induced cytokine production required residues in the CR1 and CR3 regions of E7 and resulted in reduced nuclear translocation and acetylation of the p65 sub-unit of NFκB. The results provide further evidence for a TLR7-independent role of imiquimod in the epithelial immune response and reinforce the ability of the HPV oncoproteins to disrupt the innate immune response, which may have important consequences for establishment of a chronic infection.

Human papillomaviruses (HPVs) are small dsDNA viruses that infect the squamous epithelial cells of the skin epidermis, oropharyngeal and anogenital mucosa. Infection with HPVs is associated with a spectrum of clinical lesions, ranging from common warts to cancers. Greater than 100 types have been identified^1^ and they are classified as either low- or high-risk, depending on the association with cancer development. High-risk HPVs are responsible for the majority of cervical cancers and a growing sub-set of head and neck squamous cell carcinomas^2^, and amongst these HPV16 is detected in the majority of cases^2^. Carcinogenesis is linked to persistent infection with a high-risk virus that may last several years. This suggests that HPVs have evolved mechanisms to evade the immune system.

Several studies have focussed on the immune evasion capabilities of HPV and have demonstrated that three of the virus encoded proteins; E5, E6 and E7 can impact upon the host’s ability to mount an effective immune response. E5 is the lesser understood of the three oncoproteins^3^,^4^. It is a viroporin^5^ that has been correlated with decreased cell surface expression of major histocompatibility complex (MHC) molecules and reduced activation of the adaptive immune response^6^. E6 and E7 co-operate to down-regulate expression of the pro-inflammatory cytokines IL-18 and IL-8^7^,^8^ and the chemo-attractants MCP-1 and MIP3α^9^,^10^. In addition, they increase expression of the anti-inflammatory molecule IL-18 binding protein (IL-18BP)^11^. As part of their immune evasion repertoire, both E6 and E7 have been shown to manipulate intracellular signalling pathways to inhibit activation of critical anti-viral transcription factors including NFκB and Interferon Regulatory Factors^12^,^13^,^14^.

Keratinocytes are the target for HPV infection. Studies have shown that keratinocytes can detect a wide range of pathogen associated molecular patterns (PAMPS) and respond by secreting a variety of cytokines and chemokines. These chemokines in turn recruit more specialised immune cells to the site of infection and initiate an immune response. The molecular basis for pathogen detection is an area of active investigation. Keratinocytes express a number of pattern recognition receptors (PRRs) including Toll-like receptors (TLRs). TLRs respond to a range of viral PAMPS including dsRNA (TLR3), hypomethylated DNA (TLR9) and ssRNA or imidazoquinoline compounds (TLR7/TLR8)^15^. The transcription factor NFκB is activated by a number of inflammatory stimuli, including those sensed by TLRs, and plays a critical role in mediating anti-viral responses by regulating the expression of pro-inflammatory cytokines and interferons (IFNs). NFκB is activated by phosphorylation and degradation of the inhibitory IκB protein, which frees NFκB heterodimers to translocate from the cytoplasm to the nucleus, where they participate in the transcription of NFκB target genes, including CCL20 and IL-6^16^,^17^. NFκB transcriptional activity is also controlled by post-translational modification of the NFκB sub-units. These include acetylation of the p65 sub-unit, which regulates the DNA-binding abilities of NFκB^18^,^19^.

Given the critical role of keratinocytes in eliciting the skin immune response to infection and the chronic nature of HPV infection, we sought to further understand the interplay between TLR signalling pathways and virus immune evasion strategies in primary human keratinocytes. We show that keratinocytes mount an inflammatory response to a variety of virus-associated TLR ligands including the putative TLR7 ligand imiquimod. Somewhat surprisingly, we find that NFκB activation in response to imiquimod is independent of TLR7 in keratinocytes. Moreover, we demonstrate that the major HPV oncoproteins E6 and E7 are able to block NFκB activation in response to imiquimod treatment and that this is mediated by changes to nuclear translocation and p65 acetylation. Together these data provide further evidence for the TLR-independent activity of imiquimod and highlight the extensive immune evasion capacity of HPV.

## Results

### Expression of HPV E6/E7 inhibits NFκB activation in response to viral PAMPs in primary keratinocytes

**Figure 1:**
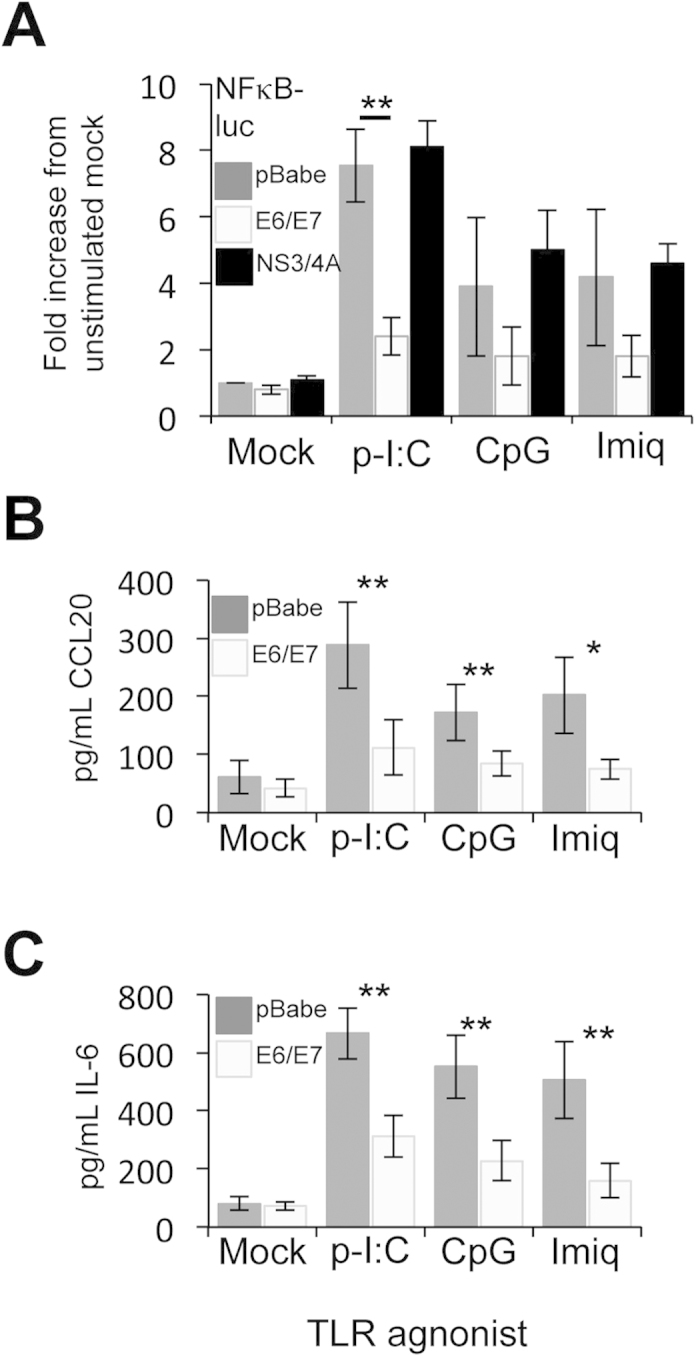
HPV16 oncoproteins disrupt TLR-mediated inflammatory signaling in primary human keratinocytes. (**A**) Primary human foreskin keratinocytes stably transduced with virus expressing pBabe-Puro empty plasmid, pBabe-Puro-16E6/E7 oncoproteins or BVDV NS3/4A as a negative control were transfected with an NFκB-dependent luciferase reporter plasmid^40^. A Renilla luciferase reporter plasmid (pRLTK) served as a transfection control. Transfected cells were subsequently treated with ligands to activate the anti-viral TLR proteins TLR3 (poly-I:C), TLR9 (CpG DNA) and TLR7 (Imiquimod) for 16 hours, before cells were lysed in Passive Lysis Buffer (Promega) and analysed using a dual luciferase system (Promega)^24^. Data are from five independent biological repeats and presented as fold increase in luciferase above the unstimulated pBabe-Puro. (**B**) CCL20 and (**C**) IL-6 protein levels in TLR-ligand stimulated cells. Cells were stimulated as in (**A**), and media was collected at 24 hours post-stimulation for analysis by ELISA for each cytokine. Data are presented as picograms of cytokine. Error bars are +/−SD and **indicates a p < 0.01, *p < 0.05, *NS* indicates not significant.

The host immune response is critical for determining clearance or persistence of HPV infection. It has now become widely accepted that in addition to skin resident immune cells, infected keratinocytes can also act as sentinels and will respond to the presence of microbial PAMPs to activate the NFκB transcription factor and drive a programme of cytokine and chemokine gene expression^20^. Despite this prominent role in initiating an immune response, their specific ability to respond to virus-associated PAMPs is not fully understood. To gain a clearer insight, we transfected primary human keratinocytes with a plasmid that drives luciferase expression from three copies of the NFκB response element^21^ and treated cells with a panel of defined TLR ligands (Fig. 1). The TLR3 ligand poly-I:C, a synthetic analogue of double stranded viral RNA, lead to the strongest activation of NFκB, as judged by levels of luciferase relative to untreated control (Fig. 1A). Keratinocytes also responded to treatment with the TLR9 ligand CpG-ODN and the imidazoquinoline imiquimod, a putative ligand for TLR7, although to a lower extent (Fig. 1A). To verify the results using a more physiologically relevant indicator, we analysed the media from mock and treated samples for levels of the chemokine CCL20 (Fig. 1B) and pro-inflammatory cytokine interleukin (IL)-6 (Fig. 1C). As NFκB-dependent gene products, their expression was increased upon stimulation with all three TLR agonists and their relative levels mirrored the data obtained using the luciferase reporter system. Expression of the major HPV16 oncoproteins, E6 and E7, is correlated with reduced activation of immune signalling pathways in a number of experimental systems. To determine whether their expression impacted upon TLR-mediated NFκB activation in a physiologically relevant setting, primary human keratinocytes stably expressing a bicistronic E6/E7 transcript were assessed in parallel to control cells. Our findings support previously published data demonstrating that HPV oncoproteins dampen the inflammatory response mediated by TLR3 and TLR9 to varying levels^22^,^23^. They also provide the first evidence that E6/E7 expression suppresses signalling emanating from a putative TLR7 ligand in keratinocytes (Fig. 1A-C). It was important to ascertain whether the results obtained may have been as a result of overexpressing exogenous proteins. To control for this keratinocytes were generated expressing the NS3/4A protease of Bovine Viral Diarrhoea Virus (BVDV), a protein previously demonstrated not to modulate innate immune signalling pathways^24^. In agreement with our previous findings, expression of NS3/4A did not change levels of NFκB-driven luciferase in response to the ligands tested (Fig. 1A). Taken together these data suggest that during a HPV infection, expression of the HPV oncoproteins would lead to an overall dampening of the innate immune response in the presence of virus PAMPS.

### Imiquimod induces an inflammatory response in primary keratinocytes independent of TLR7 expression

**Figure 2:**
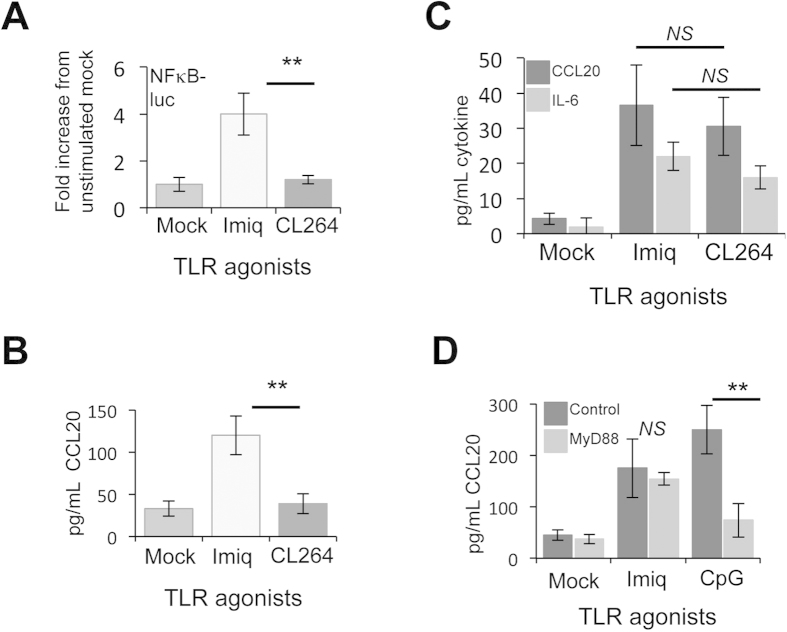
Imiquimod mediated NFκB activation and inflammatory cytokine production is independent of TLR7 in keratinocytes. (**A**) Primary human foreskin keratinocytes were transfected with an NFκB-dependent luciferase reporter plasmid. A Renilla luciferase reporter plasmid (pRLTK) served as a transfection control. Transfected cells were subsequently treated with the TLR7 ligands Imiquimod and CL264 for 16 hours, before cells were lysed in Passive Lysis Buffer (Promega) and analysed using a dual luciferase system (Promega). Data are from three independent biological repeats and presented as fold increase in luciferase above the unstimulated control. (**B**) Cells were stimulated as in (**A**), and media was collected at 24 hours post-stimulation for analysis by ELISA for CCL20 production. (**C**) Imiquimod and CL264 activate NFκB-dependent transcription in a HEK293-TLR7 cell line. HEK293-TLR7 cells were transfected, stimulated and analysed as in (**B**) for CCL20 and IL-6. (**D**) Imiquimod-mediated cytokine production is MyD88-independent in keratinocytes. Keratinocytes were treated with a MyD88 inhibitory peptide or a control peptide prior to stimulation with Imiquimod or CpG ODN. Media was collected after 24 hours and analysed for secreted CCL20 by ELISA. Data are presented as picograms of cytokine. Error bars are +/−SD. **p < 0.01 and *NS* indicates not significant.

Studies demonstrate that imidazoquinoline molecules exert their inflammatory actions through direct activation of signalling through TLR7 or TLR8^25^,^26^ present on professional antigen presenting cells including dendritic and Langerhans cells^27^. However, recent findings suggest that imiquimod may have a more direct effect on epithelial cells^28^. This additional action has been suggested to occur independently of TLR7/TLR8 receptors^28^. To address this point we undertook a high-resolution analysis to uncover the transcriptional profiles of each of the TLR present in primary human keratinocytes (Table 1). The deep RNA sequencing technique (RNA-seq) permits the identification of multiple transcripts in a single sample in an unbiased manner and can also be adapted to give quantitative data on transcript abundance. Accordingly, total polyadenylated cellular RNA was isolated from undifferentiated human foreskin keratinocytes, reverse transcribed and total cDNAs were subjected to Illumina deep sequencing. The resulting short reads were processed and aligned to the human genome using the Integrated Genome Viewer. Quantitative data on the abundance of individual transcripts was obtained by normalising the number of reads to the length of the gene i.e. using reads per kilobase (of the gene) per million mapped reads (RPKM), a value indicative of the transcript’s abundance. Using this approach, transcripts were detected that aligned with TLR1, TLR2, TLR3, TLR5, TLR6, TLR9 and TLR10 although they were of relatively low abundance compared to the highly-expressed β_2_-microglobulin transcript. We did not detect any reads (and therefore transcripts) corresponding to TLR4, TLR7 or TLR8. Thus our results demonstrating the effects of imiquimod treatment on NFκB activation and secretion of the cytokines IL-6 and CCL20 in primary keratinocytes indicated an activity beyond TLR7 or TLR8. To validate these findings, keratinocytes were treated with imiquimod or an additional TLR7 ligand called CL264. CL264 is a 9-benzyl-8 hydroxyadenine derivative shown to trigger NFκB activation at significantly lower concentrations than imiquimod. In these assays imiquimod but not CL264 was able to induce NFκB-driven luciferase expression (Fig. 2A) and this was complimented with increases in CCL20 secretion from treated cells (Fig. 2B). To verify that CL264 was functional, HEK293 cells stably expressing TLR7 were incubated with imiquimod and CL264. In contrast to primary keratinocytes both ligands induced secretion of CCL20 and IL-6 into the cell media (Fig. 2C). Finally, to underscore the TLR-independent nature of the actions of imiquimod in this system, keratinocytes were pre-treated with a 26 amino acid peptide that blocks dimerization of the critical TLR adaptor protein Myeloid Differentiation primary response gene 88 (MyD88)^29^. As TLR7 signals through MyD88, addition of this peptide would be expected to block any TLR-mediating signalling event in response to imiquimod treatment. As can be seen, addition of the MyD88 blocking peptide had no significant effect on imiquimod induced CCL20 secretion but was able to effectively prevent CCL20 secretion in response to CpG-DNA, a TLR9 ligand, which signals through MyD88 (Fig. 2D). Taken together these data indicate that primary human keratinocytes mount an inflammatory response to imiquimod that is independent of the canonical imiquimod receptor, TLR7.

### HPV E7 negatively regulates the imiquimod-induced inflammatory response in primary keratinocytes

**Figure 3:**
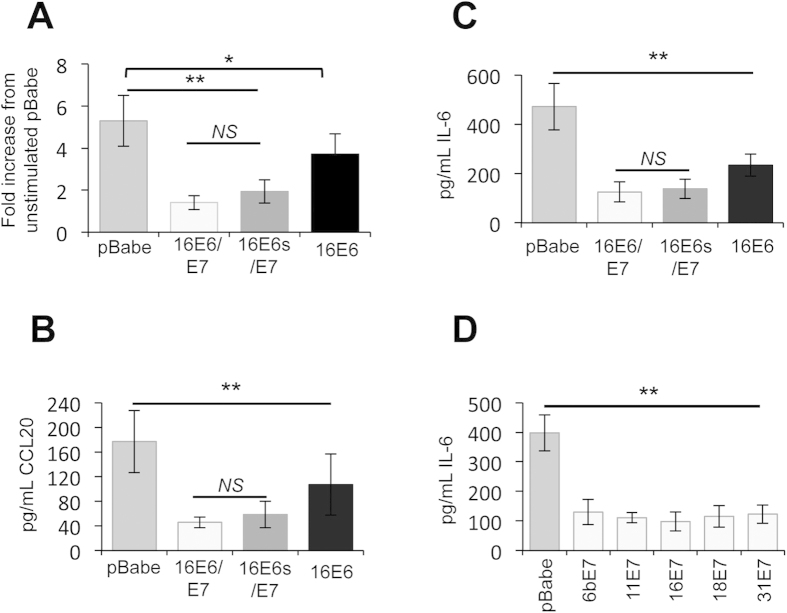
HPV E7 proteins are potent inhibitors of NFκB-dependent transcription and cytokine production in keratinocytes. (**A**) Primary human foreskin keratinocytes stably transduced with virus expressing pBabe-Puro empty plasmid or pBabe-Puro-16E6/E7 oncoproteins were transfected with an NFκB-dependent luciferase reporter plasmid. A Renilla luciferase reporter plasmid (pRLTK) served as a transfection control. Transfected cells were subsequently treated with Imiquimod for 16 hours, before cells were lysed in Passive Lysis Buffer (Promega) and analysed using a dual luciferase system (Promega). Data are from five independent biological repeats and presented as fold change compared to unstimulated pBabe-Puro. (**B**) CCL20 and (**C**) IL-6 protein levels in Imiquimod stimulated cells. Cells were stimulated as in (**A**), and media was collected at 24 hours post-stimulation for analysis by ELISA for each cytokine. (**D**) E7 from multiple HPV types inhibits Imiquimod-induced cytokine expression. Primary human foreskin keratinocytes stably expressing E7 from multiple HPV types were stimulated with Imiquimod for 24 hours and levels of secreted IL-6 analysed by ELISA. Data are presented as picograms of cytokine. Error bars are +/−SD. **p < 0.01, *p < 0.05.

To determine which of the two major HPV oncoproteins was able to prevent an inflammatory response to imiquimod treatment, keratinocytes were infected with retroviruses expressing HPV16 E6 and E7 in isolation or E6/E7 as a bicistronic transcript. Keratinocytes harbouring plasmid vector alone (mock) displayed an induction of NFκB-driven luciferase in response to treatment with imiquimod (Fig. 3A), which was decreased in the presence of 16E6. Expression of 16E7 had the most pronounced impact on imiquimod-mediated NFκB activation compared with control, which was not significantly further decreased by the co-expression of 16E6. These results were reflected in the levels of CCL20 and IL-6 secreted into the media of treated cells (Fig. 3B,C). These data suggest that whilst both oncoproteins can target the pro-inflammatory pathway, E7 is the major inhibitor of imiquimod-induced inflammatory responses in primary keratinocytes.

### E7 proteins from other HPV types inhibit NFκB activation in response to imiquimod

To establish whether the inhibition of NFκB activation was a general feature of E7 proteins, keratinocytes stably expressing E7 proteins from several mucosal types were treated with imiquimod and the levels of secreted IL-6 measured by ELISA (Fig. 3D). E7 from both low-risk (HPV6b, HPV11) and high-risk (HPV16, HPV18, HPV31) types prevented IL-6 production to comparable levels. These experiments illustrate that NFκB is a conserved target for E7 proteins of multiple HPV types, suggesting that inhibition of this inflammatory transcription factor may be necessary during the HPV life cycle rather than associated directly with transformation.

### Mapping the regions of E7 necessary to inhibit NFκB activation in response to imiquimod

**Figure 4:**
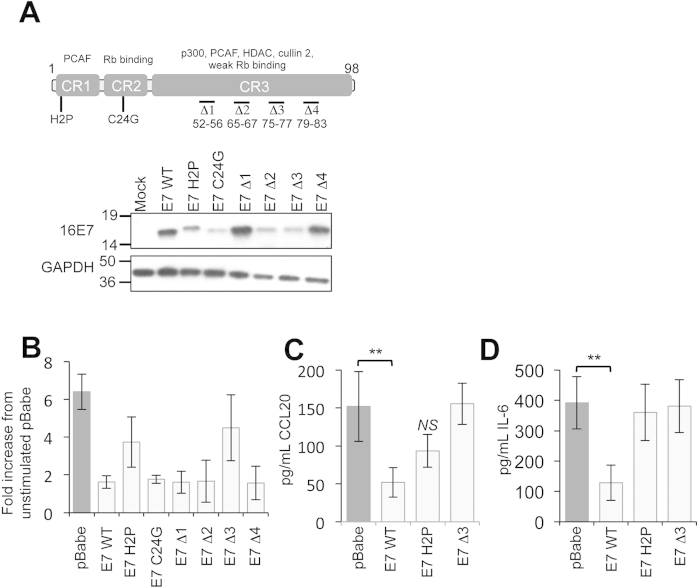
Residues in CR1 and CR3 regions of E7 are necessary for inhibition of NFκB in response to imiquimod treatment. **(A**) Schematic of E7 and the mutations used in this study. Functional regions of E7 are highlighted. Substitutions are shown, and small deletions are indicated by “Δ”. Cell lysates from representative experiments were subjected to SDS-PAGE and probed with the indicated antibodies to confirm E7 expression. GAPDH was used to demonstrate equal loading of lysates. (**B**) Primary human foreskin keratinocytes stably expressing empty plasmid (pBabe), 16E7, 16E7 H2P, 16E7 C24G, and four carboxyl-terminal truncations designated Δ1 (with deletion of amino acids 52 to 56), Δ2 (with deletion of amino acids 65 to 67), Δ3 (with deletion of amino acids 75 to 77), and Δ4 (with deletion of amino acids 79 to 83) were treated with imiquimod for 16 h and assayed as described in Fig. 3. (**C**) CCL20 and (**D**) IL-6 protein levels in Imiquimod stimulated cells in mock, E7 wild type, E7 H2P and E7Δ3 expressing cells. Cells were stimulated as in (**B**), and media was collected at 24 hours post-stimulation for analysis by ELISA for each cytokine. Data are presented as picograms of cytokine. Error bars are +/−SD. **p < 0.01 and *NS* indicates not significant.

Primary keratinocytes were transduced with retroviruses expressing E7 proteins containing mutations or small internal deletions in the amino terminal disordered conserved regions CR1 and CR2 or in the carboxyl-terminal region CR3^11^,^30^. Figure 4A shows a schematic of the proteins examined in this study. E7 proteins containing a point mutation in the LxCxE motif (C24G) or three of the four short internal deletions in CR3 (∆1 (amino acids 52–56), ∆2 (amino acids 65–67) and ∆4 (amino acids 79–83)) inhibited NFκB activation to wild type levels (Fig. 4B). In contrast E7 proteins with a mutation in the second amino acid (H2P) or the ∆3 (amino acids amino acids 75-77) deletion in CR3 showed a reduced ability to inhibit NFκB transactivation (Fig. 4B). This failure to prevent NFκB activation was further evidenced by the near wild type levels of CCL20 and IL-6 secreted from cells harbouring these mutants in response to imiquimod stimulation (Fig. 4C,D). It is plausible that the alteration or deletion of amino acids in E7 had impacted upon their overall folding or stability. Western blot confirmed stable expression of all proteins in keratinocytes, albeit at variable levels (Fig. 4A). Furthermore, previous work had demonstrated that all of the CR3 mutants retained the ability to induce pRb degradation^11^, suggesting no gross changes in E7 structure. These data suggest that the beginning of CR1 (H2P) and a short region in CR3 are required for the inhibition of NFκB in response to imiquimod.

### E7 negatively regulates imiquimod-induced activation of NFκB by reducing its nuclear translocation and acetylation

**Figure 5:**
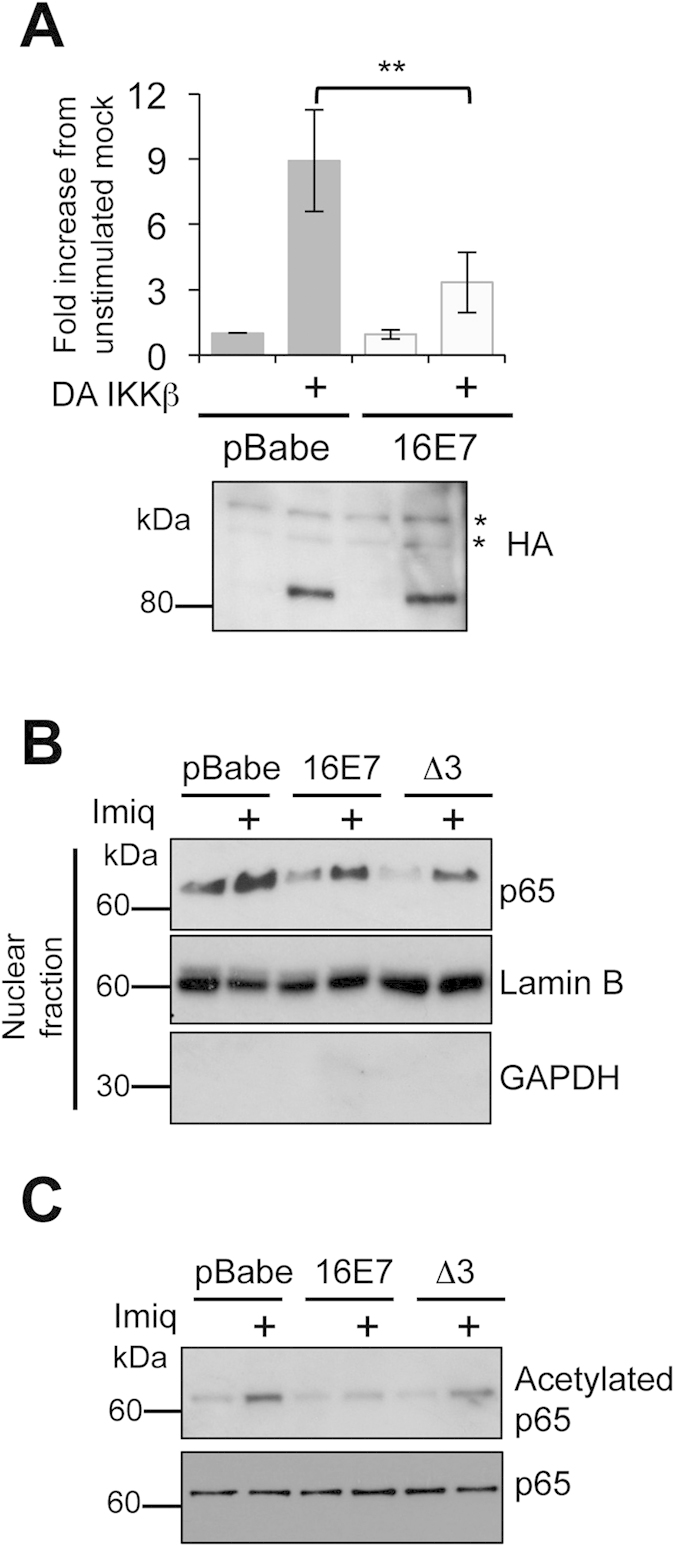
Nuclear translocation and p65 acetylation are reduced in E7 expressing cells. (**A**) Keratinocytes expressing empty plasmid (pBabe) or E7 wildtype were transfected with an NFκB-dependent luciferase reporter plasmid, Renilla luciferase transfection control together with DA HA-tagged IKKβ, and NFκB-driven transcription analysed as in Fig. 2. Error bars are +/−SD. And **indicates p < 0.01. Expression of HA-IKKβ was confirmed by western blot. *denotes a non-specific band detected by the HA antibody. (**B**) Keratinocyte lines were mock or imiquimod treated and the nuclear fractions extracted with probed with antibodies against p65. The integrity of the nuclear fraction was determined by probing for markers of the cytoplasm (GAPDH) and nucleus (Lamin B). Representative gels are shown from 3 biological repeats. (**C)** Total keratinocyte lysates from mock and imiquimod treated cells were probed with antibodies that detect total p65 and p65 acetylated at K311. Representative gels are shown from a total of 5 biological repeats. All electrophoresis gels were run under the same experimental conditions.

We sought to determine how E7 inhibits NFκB-dependent activation in response to imiquimod treatment. We addressed whether E7 negatively regulates imiquimod-induced NFκB activation by acting upstream or downstream of the critical IKK complex, which plays a key role in the activation of NFκB in response to a number of stimuli. To this end, keratinocytes stably harbouring empty plasmid or 16E7 were transfected with a constitutive active form of IKKβ in conjunction with the NFκB-driven luciferase reporter construct. As shown in Fig. 5A, IKKβ increased NFκB-driven luciferase levels above an empty plasmid control. Cells expressing E7 retained significantly decreased levels of luciferase activity, despite equal expression of the DA-IKKβ as determined by western blot. This result suggests that E7 negatively regulates imiquimod-induced NFκB activation by acting downstream of IKKβ. To test whether impaired NFκB transactivation was caused as a result of reduced nuclear import we stimulated control, wild-type E7 and E7∆3 expressing keratinocytes with imiquimod and enriched for the nuclear fraction. Samples were probed with antibodies directed against GAPDH (cytoplasmic) and Lamin B (nuclear) to verify the efficacy of the fractionation (Fig. 5B). Low levels of endogenous p65 were detected in the nuclear fractions of untreated cells (Fig. 5B). By comparison, imiquimod treatment caused an increase in p65 levels in the nuclear fraction of control cells. In agreement with a previous report^31^, expression of wild-type E7 reduced p65 nuclear import. The E7∆3 mutant caused a similar reduction in p65 nuclear import implying that the CR3 region of E7 is not necessary for this function.

In addition to nuclear shuttling, post-translational modifications, particularly protein acetylation, play a critical role in regulating NFκB activity by enhancing the DNA-binding ability of p65 for the κB site^32^,^19^. Since E7 has previously been shown to interact with the pCAF acetyltransferase^7^,^33^, we hypothesised that an additional mechanism by which E7 may inhibit NFκB activity, may involve preventing the acetylation of p65. To test this, control and E7 expressing keratinocytes were subjected to imiquimod treatment and levels of p65 acetylation determined by western blot with an acetylation specific antibody. Imiquimod induced acetylation at lysine 310 (K310) was markedly reduced in E7 expressing cells compared to control (Fig. 5C). In cells expressing E7∆3 levels of p65 acetylation were similar to the control, suggesting that E7 was no longer able to prevent this critical post-translational modification event.

## Discussion

Keratinocytes play a critical role as sentinels of the immune system. They can function as instigators of epithelial inflammation and are able to sense the presence of invading microorganisms, including viruses, as they encode a repertoire of PRRs. Keratinocytes are also important producers of antimicrobial molecules. Detection of virus encoded PAMPS culminates in the secretion of a milieu of cytokines that serve to activate and recruit key effector cells of the skin immune response. Despite this clear role for keratinocytes as instigators of the skin immune response, there are still many unresolved questions concerning the molecular basis for pathogen detection, intracellular signalling events in the infected keratinocyte and cellular interactions in the epidermis. Epitheliotropic viruses that establish chronic infections have evolved complex mechanisms to evade and subvert the skin immune response. In this regard, there is considerable literature to suggest that HPV utilises several strategies to dampen the host immune response.

We sought to address some of these outstanding issues by treating primary keratinocytes with a panel of ligands known to stimulate anti-viral PRRs and to determine whether HPV encoded oncoproteins were able to inhibit the inflammatory response. We show that E6 and E7 inhibited the activity of the transcription factor NFκB in response to a range of anti-viral PRR ligands including the putative TLR7 ligand imiquimod. As a consequence, keratinocytes secreted reduced amounts of NFκB-dependent cytokines including IL-6 and the chemo-attractant CCL20. Reduced CCL20 secretion would have a detrimental effect on the ability of keratinocytes to recruit circulating antigen presenting cells to the site of infection. Of note, HPV infected skin lesions demonstrate significant reductions both in CCL20 expression and the number of infiltrating Langerhans cells compared to uninfected controls^9^,^34^. Reduced migration of effector cells to the site of virus infection would enable HPV to evade local detection by the immune system and contribute towards its ability to persist in the infected host.

Whilst there is previous evidence for inhibition of TLR3 and TLR9 signalling by HPV oncoproteins, less is understood regarding potential evasion of TLR7 mediated immunity in keratinocytes. We demonstrated that both E6 and E7 were able to suppress NFκB activation in response to imiquimod, although levels were reduced more in the presence of E7. There is controversy surrounding the role of TLR7 in activating the skin immune response to compounds including imiquimod. It has been proposed that the anti-viral properties of imiquimod are mediated by activation of TLR7 or TLR8 in skin infiltrating mononuclear cells^27^. However, isolated keratinocytes mount a robust inflammatory response to imiquimod treatment. In addition, it has been reported that undifferentiated keratinocytes do not express TLR7 or TLR8 and that imiquimod instead exert its effects on this population of cells through an alternative pathway, which may require adenosine receptors^28^. To investigate this, we used RNA-seq to study the transcriptome of primary keratinocytes. This allowed us to study cellular gene expression in great depth and with high specificity. The results of the sequencing analysis confirmed that undifferentiated primary keratinocytes do not express TLR4, TLR7 or TLR8. We verified these data using alternative TLR7 ligands, which were unable to induce cytokine production in primary keratinocytes but were capable of stimulating HEK293 cells stably expressing TLR7. Moreover, a small peptide inhibitor of MyD88 signalling also failed to prevent to imiquimod induced cytokine secretion in keratinocytes but was able to effectively block TLR9 mediated signalling. Similar findings have also been shown with imiquimod treated splenocytes from MyD88-deficient mice^28^.

This unidentified pathway induced NFκB activation in response to imiquimod treatment as evidenced by reporter assays and resulted in the production of NFκB-dependent cytokine production (IL-6 and CCL20). Interestingly, this pathway was also effectively inhibited by E7 expression. A panel of established E7 mutants was used to map the functional requirements for NFκB inhibition^11^,^35^. We found that E7 proteins containing a point mutation in the CR1 region (H2P) or a small internal deletion in CR3 (amino acids 75–77) were unable to repress NFκB activation to the same level as wildtype E7 and secreted cytokine levels similar to the control samples. One mechanism by which E7 perturbs imiquimod signalling might be by preventing the nuclear entry of NFκB sub-units^31^. We confirmed that E7 partially suppresses p65 nuclear translocation and that the CR3 mutant that is unable to inhibit NFκB activation to control levels was still able to impair nuclear translocation. These data suggested that E7 might prevent NFκB activation by additional mechanisms. For example H2 and CR3 regions might be required to interact with cellular proteins that are essential components of the NFκB signalling pathway. In this regard, previous studies have linked an interaction between E7 and the histone deacetylase P/CAF with suppression of host cell transcription^7^. NFκB-dependent transcription requires multiple co-activator proteins, including histone acetyltransferases such as P/CAF^18^. In particular acetylation of lysine residues including K310 on the p65 sub-unit are essential for effective DNA binding by NFκB^18^. Using acetylation specific antibodies we demonstrated that p65 acetylation was reduced in imiquimod treated cells expressing E7. These data suggest that E7 may interfere with histone acetyltransferase activity to suppress NFκB activation. In support of this, mutation of the second amino acid of E7 (H2P) reduced the binding of E7 to P/CAF^7^. In our assays this mutant was unable to suppress NFκB activation and cytokine secretion in response to imiquimod. Whilst it is currently not known if the E7∆3 can bind P/CAF, it should be noted that a second study identified a point mutation in the CR3 region of E7 that ablated binding to P/CAF in a yeast-2-hybrid assay^33^. Together, these data suggest that multiple regions of E7 may be required for the interaction with P/CAF.

From several studies and our data it can be concluded that keratinocytes respond to imiquimod through at least two pathways. One that requires TLR7 and may be active in differentiated keratinocytes^36^, and a second, poorly characterised, pathway in undifferentiated cells that lack TLR7 expression. By targeting NFκB signalling, E7 effectively suppresses the anti-viral response to activation of either pathway. Our study now adds a further example of E7 immune evasion and suggests that expression of HPV oncoproteins provides a broad spectrum dampening of the cytokine response from infected keratinocytes that would suppress the recruitment of effector cells to the site of infection and help explain HPV persistence in the epidermis. Furthermore, our findings are of importance for clinical treatment of epithelial infections and lesions since the inflammatory response of topical imiquimod application seems to be closely linked to the therapeutic effect. It has not yet been deciphered to what extent the efficacy of imiquimod relies on the epidermal response. Therefore, to further optimise treatment effect to side effect ratio and efficacy a better understanding of the response pathways to imiquimod will impact further pharmacotherapy developments.

## Materials and Methods

### Cell lines and viruses

Primary human foreskin keratinocytes were cultured as described^37^. Cells were grown in SFM keratinocyte growth media (Invitrogen) containing 25 mg/mL bovine pituitary extract and 0.1 ng/mL EGF. Cells were passaged for a maximum of 6 passages. Stocks of retrovirus for transduction of keratinocytes were produced by transfection of the Phoenix packing cell line. Supernatants from transfected cells were added to target cells with 5 μg/mL polybrene at 37 °C. Virus was removed from cells after 6 hours incubation and cells were fed fresh media. After 48 hours recovery at 37 °C cells were placed under selection in 5 μg/mL puromycin.

### Plasmids

The plasmid pBabe-Puro was used to transiently or stably express E6 or E6/E7 products. Constructs containing E6, E7 or E6/E7 from HPV6, 11, 16 and 31 were provided by Prof. Dennis McCance (Queens, University Belfast) and were previously described^7^. HPV18 E7 sequences were PCR amplified from full-length HPV18 genome^37^ and ligated into pBabe-Puro using BamHI and EcoRI sites. HPV16 E7 point and truncation mutants used were previously described^30^. All plasmid inserts were sequenced using GATC.

### Dual luciferase assays

Dual luciferase reporter assays were performed as previously described^21^,^24^. Keratinocytes were seeded at 3 × 10^5^ cells/mL in 24 well dishes the day prior to transfection. Cells were transfected with NFκB-driven firefly luciferase reporter, Renilla luciferase (RLTK) expressing plasmid as transfection control, and each of the plasmids encoding HPV proteins using the PEI reagent (PolySciences). At 24 hours post transfection cells were stimulated with the appropriate reagents for 6–24 hours (see figure legends for details – all ligands purchased from Invivogen) prior to lysis in passive lysis buffer (Promega). Firefly luciferase levels were normalised to Renilla levels and fold induction values were calculated relative to the normalised activity of the mock.

### CCL20 and IL-6 cytokine assays

Quantities of CCL20 and IL-6 in cell-free supernatants were determined using the R&D systems DuoSet ELISA kit according to manufacturer’s instructions.

### Nuclear fractionation

Control and stimulated cells grown in 10 cm dishes were lysed in cytoplasmic lysis buffer (20 mM Tris-HCl pH 7.4, 100 mM NaCl, 5 mM MgCl_2_, 0.5% NP40 and protease inhibitors) by incubation on ice for 30 min with frequent vortexing. The supernatant accounted for the cytoplasmic fraction. The nuclear pellet was washed twice in cytoplasmic lysis buffer to reduce cytoplasmic contamination before resuspension in radio-immunoprecipitation (RIPA) buffer. Debris was removed by centrifugation. Samples were then subject to analysis by western blot. Lamin B1 (Calbiochem) and GAPDH (Abcam) acted as markers of the nuclear and cytoplasmic fractions.

### Western blotting

Primary keratinocytes were stimulated with the appropriate ligand for the time indicated in the legend. Media was aspirated and cells washed with ice cold PBS and harvested in Dundee lysis buffer^38^. Lysates were analysed by SDS PAGE and probed with antibodies against p65, acetylated p65 (Cell Signalling Technology), Lamin B (Calbiochem) and GAPDH (Abcam). Secondary antibodies used were HRP-conjugated anti-mouse and anti-rabbit antibodies from Sigma. Blots were developed using enhanced chemiluminescence (ECL) reagents (Pierce).

### Transcriptome analysis

Total RNA was isolated from cell cultures (approx 3 × 10^6^ cells) using TRIzol reagent (Invitrogen, USA) following the manufacturer’s instructions^11^. Any contaminating genomic DNA was removed by incubation with 5U of RNase-free DNase (Promega, UK). Further purification of mRNA from total RNA was performed using the Dynabead purification system (Invitrogen, USA) and eluted into DEPC-treated H_2_O. Double-stranded cDNA was synthesised using the SuperScript II double-stranded cDNA synthesis kit (Invitrogen, USA). This was purified from the reaction mix using the QIAquick DNA extraction system (Qiagen, USA) before quantification by the Quanti-PicoGreen assay (Invitrogen, USA) and POLARstar Optima (Optima Scientific, UK). Fractionation of double-stranded cDNA to 200–300 bp fragments was performed using the Covaris S instrument (Covaris, USA) before end-repair was performed using the Paired-end sequence sample preparation kit (Illumina, USA). Adapter molecules were ligated to the cDNA molecules following the protocol of the Paired-End sequence sample preparation kit (Illumina, USA).

Next-generation sequencing of the ds-cDNA from primary human keratinocytes was performed using the Illumina Genome Analyser II (Illumina, USA) following the manufacturer’s recommendations in a single-strand manner. Sequencing was performed over 85 cycles with a mean of 43 million reads per sample. The .qseq sequence output from the Illumina genome analyser II was converted to the FASTQ format before initial screening for the removal of the adapter sequences and PCR artefacts from the reads using the FASTX-Toolkit v0.0.13.

FASTQ files generated by next generation sequencing on the Illumina platform were uploaded to the Galaxy analysis platform for the alignment to genomes of interest^39^. Initially FASTQ formatted files were converted to FASTQ-Sanger format and analysed for quality across the read length. When transcript quality across the read fell below a threshold level the read was trimmed to remove bases of poor quality. Read quality varied between samples resulting in different trimming requirements for each sample. The trimmed reads were aligned to the human genome using TopHat 1.3.0 program for *de novo* RNA-seq transcript assembly, with a two nucleotide base error incorporation per read (excluding the anchor region). This method enabled accurate analysis of gene expression in the analysed samples. The .bam files generated by TopHat were indexed before profiling and manual analysis using the Integrated Genome Viewer 2.0 (Broad Institute, USA).

## Additional Information

**How to cite this article**: Richards, K. H. *et al.* The human papillomavirus (HPV) E7 protein antagonises an Imiquimod-induced inflammatory pathway in primary human keratinocytes. *Sci. Rep.* **5**, 12922; doi: 10.1038/srep12922 (2015).

## Figures and Tables

**Table 1:** TLR expression profile in primary keratinocytes.

Transcript	RPKM
Beta-2-microglobulin	26.54
TLR1	0.05
TLR2	0.16
TLR3	0.09
TLR4	0
TLR5	0.05
TLR6	0.56
TLR7	0
TLR8	0
TLR9	0.05
TLR10	0.01

Levels of TLR mRNAs in primary human keratinocytes were quantified from RNA-Seq data using the Cufflinks program with output expressed as reads per kilobase per million mapped reads (RKPM). This program generates normalised data that is proportional to the level of the transcript in the original population of cellular mRNAs. The levels of β_2_-microglobulin (B2M) transcripts were included as a positive control.
